# Comparison of hemoglobin A1c goal achievement with the addition of pioglitazone to maximal/highest tolerated doses of sulfonylurea and metformin combination therapy

**DOI:** 10.3109/21556660.2012.707996

**Published:** 2012-06-29

**Authors:** M. Shawn McFarland, Lana Huddleston, Kumar Tammareddi, Michael McKenzie, Jennifer Bean

**Affiliations:** 1 Department of Veterans Affairs – TN Valley Health Care System, Pharmacy Service, Murfreesboro, TN; The University of Tennessee College of Pharmacy, Memphis, TNUSA; 2 Department of Veterans Affairs – TN Valley Health Care System, Pharmacy Service, Nashville, TNUSA; 3 Department of Veterans Affairs – TN Valley Healthcare System, Murfreesboro, TNUSA; 4 The University of Tennessee Department of Clinical Pharmacy, Memphis, TNUSA; 5 Department of Veterans Affairs – TN Valley Healthcare System, Department of Pharmacy, Murfreesboro, TN; The University of Tennessee Department of Clinical Pharmacy, Memphis, TNUSA

**Keywords:** Hemoglobin A1c, Thiazolidinediones, Type 2 diabetes

## Abstract

**Objectives:**

It has been proposed that the combination of thiazolidinedione (TZD) therapy to metformin and sulfonylurea is beneficial due to each medication having a unique mechanism of action. Within the Veterans Affairs Hospital, specific criteria of use define when TZD therapy can be initiated. Most patients who receive TZD therapy have failed other medications prior to use. The primary objective of this study was to determine the percentage of patients achieving the American Diabetes Association (ADA) goal hemoglobin A1c (A1c) of less than 7% with the addition of pioglitazone to the maximal/highest tolerated doses of sulfonylurea and metformin combination therapy.

**Methods:**

This was a six healthcare system retrospective, descriptive, analysis of type 2 diabetic patients (DM-2). Patients must have received the maximal/highest tolerated doses of sulfonylurea and metformin combination therapy and have been TZD naïve or off TZD therapy for a minimum of 6 months, a baseline A1c greater than 7%, a repeat A1c at 3 and 6 months available, and deemed medication compliant.

**Results:**

We evaluated 98 total patients. The percentage of veteran patients achieving ADA goal A1c of less than 7% after the addition of pioglitazone reached statistical significance at both 3 and 6 months post TZD initiation. The mean reduction in A1c post-pioglitazone initiation was 0.67% (SD ± 0.92) and 0.78% (SD ± 0.94) at 3 and 6 months, respectively.

**Conclusion:**

The addition of pioglitazone to veteran patients who were already receiving maximal/highest tolerated doses of sulfonylurea was able to achieve a higher percentage in with the ADA goal A1c of less than 7%. Initiating pioglitazone in patients with an A1c of 9% or greater did not reach statistical significance in achieving an A1c less than 7%. The initial starting dose of pioglitazone 30 mg can be considered as compared to 15 mg daily if contraindications do not exist.

## Introduction

Type 2 diabetes mellitus is a chronic, progressive condition that affects approximately 23.6 million children and adults in the United States. Complications associated with diabetes include heart disease, stroke, hypertension, kidney disease, amputation, and death^1^. Proper control of blood glucose is essential and has been shown to reduce and delay the progression of micro- and macrovascular complications associated with type 2 diabetes. The United Kingdom Prospective Diabetes Study showed that achieving a hemoglobin A1c (A1c) of less than 7% reduces the risk of diabetic complications, especially microvascular complications^2^.

Insulin resistance is considered a core metabolic defect in type 2 diabetic patients (DM-2). Along with insulin resistance and increased hepatic gluconeogenisis, over time DM-2 have a progressive loss of beta cell function that leads to insulin deficiency and a resultant hyperglycemic state. Medications such as sulfonylureas, metformin, and thiazolidinediones (TZDs) target these specific defects. Sulfonylureas are associated with the promotion of insulin secretion, metformin the inhibition of hepatic gluconeogenesis, and TZDs increased insulin sensitivity of the liver, fat, and muscle by agonist activity at the PPARγ receptor.

A 2009 consensus statement from the American Diabetes Association (ADA) and the European Association for the Study of Diabetes recommended a step-wise approach to the management of DM-2 with metformin being the recommended first-line agent in the treatment algorithm^3^. As additional treatment is required, addition of either sulfonylurea or basal insulin are preferred with TZDs being considered tier 2 agents for combination therapy with either metformin alone or with the combination of metformin and sulfonylurea therapy.

It has been proposed that the combination of TZD therapy to metformin and sulfonylurea is beneficial due to each medication having a unique mechanism of action. In a study by Aljabri *et al*., the effects of the addition of pioglitazone versus bedtime insulin to maximal doses of metformin and sulfonylurea resulted in a reduction of A1c at study end of −1.9 (SD ± 1.5) and −2.3 (SD ± 1.5), respectively (*p* = 0.32)^4^. However, only 23% of those in the pioglitazone group and 21% in the insulin group achieved the ADA stated A1c goal of less than 7%. In a study by Scheen *et al*., the long term glycemic control with metformin-sulfonylurea-pioglitazone triple therapy was assessed^5^. Approximately 46.4% of patients achieved the ADA target A1c of less than 7% with the addition of pioglitazone therapy. In contrast, patients in this particular study had a baseline A1c of 8.2% while those in the Aljabri study had a baseline value of 9.7%.

Medications for diabetes management on the Veterans Affairs (VA) national formulary include sulfonylureas (glipizide, glyburide), a biguanide (metformin), and insulins (aspart, glargine, regular, NPH, 70/30). Other antidiabetic medications, including TZDs, require non-formulary consults. TZDs, pioglitazone specifically, have defined criteria for use. To qualify for TZD treatment the following must be met: (1) contraindication to metformin or sulfonylureas, (2) inadequate glycemic control on monotherapy metformin or sulfonylurea or dual therapy with these agents, and (3) patient is not a good candidate or refuses addition of insulin. Within Veteran Integrated Service Network-9 (VISN-9), specific non-formulary reviewers evaluated the criteria of use for TZD and either approve or disapprove initiation via a formal consult process.

Given the criteria for initiation of TZDs within the VA system predisposes use to patients with contraindications or inadequate response to the combination of first line agents metformin and sulfonylurea and given the achievement of the ADA A1c goal of less than 7% is not known in this population, we proposed a study to evaluate the addition of a TZD to a regimen that consisted of maximized metformin and sulfonylurea in regards to obtainment of a goal A1c of less than 7% in a veteran population compared to previously reported literature in a non veteran population.

## Patients and methods

This was a six healthcare systems, retrospective, descriptive, analysis of diabetic patients treated within the veteran integrated service network-9 (VISN-9). The Department of Veterans Affairs (VA) MidSouth Healthcare Network (VISN-9) is an integrated healthcare delivery system comprised of five Joint Commission accredited medical centers and one Joint Commission accredited Healthcare System. This study was approved by the VA-Tennessee Valley Healthcare System Institutional Review Board.

Patients were selected based upon their diabetic medication regimen. Patients must have received the maximal/highest tolerated doses of sulfonylurea (glyburide, glipizide, glimepiride) and metformin combination therapy and have been managed within the VISN-9 healthcare system between the time frame of May 2007 to March 2010. Maximal highest dose of sulfonylurea was defined as what dose of the medications the patient was on when the TZD was approved for use. Patients must have been TZD naïve or off TZD therapy for a minimum of 6 months, had an A1c baseline value greater than 7% prior to TZD initiation, and have had repeat A1c values at 3 months (±45 days) and 6 months (±45 days) post TZD initiation. Patients must have been adherent to their diabetic medication regimen.

Patients were excluded if they had type 1 diabetes mellitus or were treated with other antidiabetic pharmacotherapy not mentioned in the inclusion criteria, had a history of active liver disease or an alanine transaminase level greater than 2.5 times the upper limit of normal, or concomitant therapy with immunosuppressants or corticosteroids that may have resulted in hyperglycemia as a side-effect.

Data was extracted electronically to include patient characteristics, laboratory values, and pharmacy data. Patient characteristics included age, race, gender, and if a specific diagnosis of liver disease existed. Laboratory data included A1c values at baseline prior to beginning TZD therapy (time 0, −60 days) and then again at three (±45 days) and 6 months (±45 days) after TZD initiation. Other laboratory data that was assessed included the evidence of elevation of 2.5 times the upper limit of normal of alanine transaminase. Pharmacy data was assessed to determine the diabetic medication regimen the patient was taking, evidence of adherence, prior TZD use, TZD dose and agents that may have resulted in hyperglycemia as a side-effect. Specifically, we evaluated the dose of TZD at initiation, 3 month and 6 months. The dose of metformin and sulfonylurea

The primary outcome of this study was to assess the percentage of patients who achieved the ADA goal A1c value of less than 7% with the addition of pioglitazone to the maximal/highest tolerated doses of sulfonylurea and metformin combination therapy compared to previous literature. In addition, secondary outcomes included: (1) the percentage in A1c reduction achieved with the addition of pioglitazone at 3 and 6 months and (2) the correlation of pioglitazone dose and A1c achieved at 3 and 6 months.

For the primary endpoint, the Fisher’s exact test was used to evaluate the achievement of ADA A1c goal of less than 7% after TZD initiation. For the secondary endpoint, percent A1c reduction, the *t*-test was used given the data was normally distributed. Normally distributed continuous variables were represented by the mean ± standard deviation. In regard to the previous literature, 23% and 46% of patients on maximal tolerated doses of metformin and sulfonylurea had an A1c of less than 7% after initiation of a TZD^4,5^. Therefore, we chose to use an average of these two findings of 34.5%. We calculated our sample size to be 35 with 90% power and an alpha of 0.05. Alpha was set at 0.05 *a priori*.

## Results

The initial cohort consisted of 1201 patients and was reduced to a final cohort of 98. Reasons for exclusion are described in Table 1. The average age of the cohort was 64 years (SD ± 7.67), with Caucasian males representing the majority of the population. Additional demographic information can be found in Table 2.

**Table 1.** Reasons for exclusion.

Reasons for exclusion Initial cohort = 1201	Number excluded
Noncompliance	261
Diagnosis of type 1 diabetes mellitus and/or liver disease	216
Use of steroids	105
Use of cyclosporine or tacrolimus	2
Initial A1c ≤ 7%	100
Use of incretin mimetic agent	4
Use of any insulin	345
Use of acarbose	127
Use of nateglinide or repaglinide	2
Use of sitagliptin or saxagliptin	10
Use of pramlintide	0
Initial AST, ALT (AST ≥ 115, ALT ≥ 172)	3
AST, ALT at 3 months (AST ≥ 115, ALT ≥ 172)	1
AST, ALT at 6 months (AST ≥ 115, ALT ≥ 172)	2
No A1c prior to pioglitazone initiation	240
No A1c at 3 months	616
No A1c at 6 months	534

AST, aspartate transaminase; ALT, alanine transaminase; A1c, hemoglobin A1c.

**Table 2.** Initial demographics.

Parameter	Number (% of patients)
Age (years and SD)	64.69 (±7.67)
Gender
Male	97 (98.98%)
Female	1 (1.02%)
Race
White	79 (80.61%)
Black	9 (9.18%)
Unknown	10 (10.20%)

The mean initial A1c for the 98 patients evaluated was 8.25% (SD ± 0.78). After initiation of pioglitazone therapy a total of 23 patients (23.4%) achieved an A1c less than 7% which was lower than previous reported literature for A1c obtainment (*p* < 0.0001). After 6 months of pioglitazone therapy, 33 patients (33.6%) achieved an A1c less than 7% (*p* < 0.0001) (see Table 3). After 3 months of pioglitazone therapy, the overall mean reduction in A1c was 0.67% (SD ± 0.92) to a mean A1c of 7.58% (SD ± 1.04). The mean A1c reduction achieved after 6 months of therapy was 0.78% (SD ± 0.94) to an achieved A1c of 7.47% (SD ± 1.08) (see Table 3). In regards to dose, more patients achieved the A1c goal and had a greater A1c reduction at 3 and 6 months with the 30-mg and 45-mg dose of pioglitazone versus the 15-mg (see Table 4). It was also noted, that the higher the A1c at baseline, fewer patients achieved the A1c goal with more patients with an A1c between 7 and 7.9% having achieved a goal A1c compared to other A1c ranges (see Table 5).

**Table 3.** Mean A1c values (initial, 3 months, 6 months).

	Mean A1c (%) (±SD)	Mean A1c reduction (%) (±SD)	Number of patients achieving an A1c <7% (% of patients)	*p*-value
Initial	8.25 (±0.78)	N/A	N/A	N/A
3 months	7.58 (±1.04)	0.67 (±0.92)	23 (23.47%)	<0.0001
6 months	7.47 (±1.08)	0.78 (±0.94)	33 (33.67%)	<0.0001

SD, standard deviation; N/A, not applicable.

**Table 4.** Correlation of initial pioglitazone dose and the mean A1c achieved at 3 and 6 months.

Initial dose of pioglitazone (mg)	Mean initial A1c (%) (±SD)	Mean A1c (%) at 3 months (±SD) Mean change in A1c (±SD) % achieving A1c <7%	Mean pioglitazone dose at 3 months in mg (±SD)	Mean A1c (%) at 6 months (±SD) Mean change in A1c (±SD) % achieving A1c <7%
15 (*n* = 29)	8.21 (±0.72)	7.84 (±0.85)	23.65 (±10.54)	7.61 (±0.67)
		0.37 (±0.63)		0.6 (±0.75)
		10 (*p* = 0.2368)		13 (*p* = 0.1120)
30 (*n* = 63)	8.26 (±0.8)	7.57 (±1.09)	32.95 (±6.01)	7.45 (±1.21)
		0.69 (±0.92)		0.8 (±0.96)
		25 (*p* < 0.0001)		42 (*p* < 0.0001)
45 (*n* = 3)	8.6 (±1.4)	7.2 (±0.52)	45 (±0)	7.73 (±0.95)
		1.4 (±1.15)		0.87 (±0.46)
		2 (*p* = 1)		0 (*p* = 1)

**Table 5.** Correlation of initial A1c range and pioglitazone dose.

Initial A1c, range (%)	Mean initial A1c (%) (±SD)	Mean initial pioglitazone dose in mg (±SD)	Mean A1c (%) at 3 months (±SD) Mean change in A1c (±SD) % achieving A1c <7%	Mean pioglitazone dose at 3 months in mg (±SD)	Mean A1c (%) at 6 months (±SD) Mean change in A1c (±SD) % achieving A1c <7%
7–7.9 (*n* = 42)	7.62 (±0.22)	24.64 (±7.99)	7.34 (±0.66)	30.41 (±10.3)	7.23 (±0.56)
			0.28 ( ±0.63)		(0.39 ± 0.56)
			26 (*p* = 0.0005)		38 (*p* < 0.0001)
8–8.9 (*n* = 39)	8.34 (±0.26)	27.31 (±7.60)	7.44 (±0.86)	31.54 (±8.28)	7.28 (±0.91)
			0.9 (±0.87)		(1.06 ± 0.95)
			25 (*p* = 0.001)		38 (*p* < 0.0001)
9–9.9 (*n* = 14)	9.41 (±0.33)	25.71 (±7.03)	8.19 (±1.25)	26.54 (±6.58)	8.11 (±1.25)
			1.21 (±1.12)		(1.3 ± 1.13)
			14 (*p* = 0.4815)		14 (*p* = 0.4815)
>10 (*n* = 3)	10.6 (±0.69)	30 (±15)	10 (±2.61)	40 (±8.66)	10.33 (±2.66)
			0.6 (±2.01)		0.27 (±1.96)
			0 (*p* = 1)		0 (*p* = 1)

## Discussion

TZDs are one antidiabetic option in the management of DM-2 that have been shown to improve glucose control as well as preserve beta cell function long term^6^. In our study, initiation of pioglitazone in 98 veterans who had already received the maximal/highest tolerated doses of sulfonylurea and metformin in combination yielded an improved percentage who achieved an A1c goal of less than 7% at both 3 and 6 months compared to previous literature. Specifically, it was noted that veterans with an initial A1c of 9% or greater reached an A1c goal less frequently than those with an initial A1c ranging from 7 to 8.9% after both 3 and 6 months of pioglitazone therapy. In comparing the veterans initiated on pioglitazone 15 mg with those initiated on pioglitazone 30 mg, those treated with 15 mg reached an A1C less than 7% at either 3 or 6 months less often.

Previous literature addressing addition of a TZD to metformin and sulfonylurea therapy have shown that achievement of an A1c less than 7% can be obtained, however the A1c prior to TZD initiation is a major factor in determining if this goal can be reached. As previously mentioned, in the Aljabri study where the mean initial A1c was 9.7%, only 23% of patients reached goal A1c when TZD therapy was added to a regimen that consisted of metformin and sulfonylurea therapy, while 21% reached goal A1c if insulin was added to their regimen. This study did not show statistical significance between adding a TZD versus insulin to these patients. Scheen and colleagues study showed that 46.4% of patients achieved goal with the combination. However, the mean initial A1c in this study was 8.2%. Our results showed similar findings in that the higher the A1c, the more difficult it was to treat patients to a goal A1c of less than 7%. Despite the discovery of therapeutic entities to aid in the treatment of type 2 diabetes, glucose control is still not achieved or maintained in most patients. Many practitioners target a goal A1c of less than 7% for their patients based on the recommendation of current guidelines in an effort to avert macrovascular and microvascular complications associated with type 2 diabetes mellitus^7^. Recent studies have questioned the effectiveness of aggressive glucose reduction in type 2 diabetes in regards to reduction of macrovascular complications^8–10^. For this reason, treatment of diabetes with multiple medications needs to be evaluated based on overall risk of complications, side-effects of the medications chosen and cost. For example, in many frail elderly patients, an A1c goal of less than 8%, as suggested by the American Geriatrics Society, may be considered versus risk of tighter glycemic control and the increased risk of either hypoglycemia or side-effects from medications used to treat diabetes^11^.

Use of TZD therapy in the VA is limited due to restrictions including the following: (1) those that have contraindications to metformin or sulfonylurea therapy, (2) those with inadequate glycemic control with metformin and/or sulfonylurea therapy, (3) those who are not good candidates or refuse insulin. These restrictions limit the use of the medications to patients who have failed metformin or sulfonylurea monotherapy or combination therapy with these agents. Our study suggests that use of TZD therapy in our population should continue to be limited to a subset of patients where goal obtainment can be achieved. Specifically, a target population where the A1c is less than 8%. TZD therapy is not without limitations. Edema, increased risk of non-vertebral fractures and now a possible link to bladder cancer have all been suggested. In addition, the cost of TZD therapy can at times limit the overall use of the therapy. Practitioners must consider the added benefit of the medication in regards to glucose reduction versus the side effects and cost of the medication.

There were several limitations to our study. The first was that it was retrospective in nature. Although we tried to address confounding factors as much as possible, it is difficult to identify all factors that go into diabetes management within the confines of a retrospective data pull. Another limitation of the study was the evaluation of compliance. Since this was a retrospective data pull we were unable to interview the patients in regard to their compliance; therefore, we chose to utilize a definition for compliance based on refill history. This definition may have skewed our findings. Also, given the average age of our patient population, some providers may have been targeting a goal A1c of less than 8% instead of less than 7%. We did not look at long term outcomes associated with reduction of A1c but specifically on just the overall reduction and obtainment of recognized goals for patients with DM-2. Of our initial data pull, we excluded 1103 patients who were prescribed a TZD. This exclusion had the potential to yield a bias to favor patients who tolerated a TZD. Upon further review, multiple patients were excluded for more than 1 reason. However, 1103 patients were excluded secondary to no A1c at 3 and 6 months. These patients would have been unable to be evaluated in regards to TZD efficacy if included. A total of 488 patients were excluded due to being on a medication other than metformin and sulfonylurea. This could be considered a limitation outside of our setting. However, for our study hypothesis specifically evaluated the benefit of adding a TZD to metformin and sulfonylurea therapy in order to determine if current formulary restrictions were applicable. One major limitation was our inability to evaluate for adverse effects associated with TZD initiation. Given our retrospective design, we and short term follow up, we did not evaluate long term complications like bladder cancer or osteopenia. We also were unable to evaluated the duration of diabetes to delineate the differences in response in newly diagnosed versus patient with a longer history of diabetes. Our patient population and the requirements for use of metformin and sulfonylurea first would lend to patients who use TZD to have a longer duration of disease. Finally, we were limited in the evaluation of medications the patient may have been taking outside the VA to those properly documented by the provider, which may have included other antidiabetic or hyperglycemic causing medications.

## Conclusion

TZD agents have been previously studied in several different clinical settings. Their current place in therapy as defined by the ADA are as tier 2 agents for use with either failure of metformin monotherapy or failure of metformin and sulfonylurea combination therapy. Based upon the results of this study, the addition of pioglitazone to veteran patients already receiving maximal/highest tolerated doses of sulfonylurea and metformin combination therapy, was shown to achieve a higher percentage of patients to the ADA goal A1c of less than 7%. However, most patients did not achieve goal A1c when the A1c prior to TZD initiation was greater than 9%. Therefore, initiation of a TZD agent in patients with an A1c of 9% or greater, would most likely not achieve the patient to goal. Pioglitazone 30 mg daily could be considered as the initial starting dose if contraindications do not exist, as patients initiated on 30 mg daily reached statistical significance at both 3 and 6 months with relation to achieving an A1c of less than 7%, while those initiated on 15-mg daily did not. It is also important for providers to determine if patients could also benefit more from other therapies.

## Transparency

### Declaration of financial/other relationships

The peer reviewers on this manuscript have disclosed that they have no relevant financial relationships.
